# Mo-Doped LaFeO_3_ Gas Sensors with Enhanced Sensing Performance for Triethylamine Gas

**DOI:** 10.3390/s24154851

**Published:** 2024-07-25

**Authors:** Chenyu Shen, Hongjian Liang, Ziyue Zhao, Suyi Guo, Yuxiang Chen, Zhenquan Tan, Xue-Zhi Song, Xiaofeng Wang

**Affiliations:** 1 School of General Education, Dalian University of Technology, 2 Dagong Road, Liaodongwan New District, Panjin 124221, China; 101@mail.dlut.edu.cn (C.S.); zdflbj@mail.dlut.edu.cn (H.L.); 1677445675@mail.dlut.edu.cn (S.G.); 2 Leicester International Institute, Dalian University of Technology, 2 Dagong Road, Liaodongwan New District, Panjin 124221, China; nmgzzy2003@mail.dlut.edu.cn (Z.Z.); tanzq@dlut.edu.cn (Z.T.); 3 School of Chemical Engineering, Ocean and Life Sciences, Dalian University of Technology, 2 Dagong Road, Liaodongwan New District, Panjin 124221, China; reche@mail.dlut.edu.cn

**Keywords:** perovskite, rare earth, metal oxide, gas sensor

## Abstract

Triethylamine is a common volatile organic compound (VOC) that plays an important role in areas such as organic solvents, chemical industries, dyestuffs, and leather treatments. However, exposure to triethylamine atmosphere can pose a serious threat to human health. In this study, gas-sensing semiconductor materials of LaFeO_3_ nano materials with different Mo-doping ratios were synthesized by the sol–gel method. The crystal structures, micro morphologies, and surface states of the prepared samples were characterized by XRD, SEM, and XPS, respectively. The gas-sensing tests showed that the Mo doping enhanced the gas-sensing performance of LaFeO_3_. Especially, the 4% Mo-doped LaFeO_3_ exhibited the highest response towards triethylamine (TEA) gas, a value approximately 11 times greater than that of pure LaFeO_3_. Meantime, the 4% Mo-doped LaFeO_3_ sensor showed a remarkably robust linear correlation between the response and the concentration (R^2^ = 0.99736). In addition, the selectivity, stability, response/recovery time, and moisture-proof properties were evaluated. Finally, the gas-sensing mechanism is discussed. This study provides an idea for exploring a new type of efficient and low-cost metal-doped LaFeO_3_ sensor to monitor the concentration of triethylamine gas for the purpose of safeguarding human health and safety.

## 1. Introduction

With the development of modern industrial products, volatile organic compounds (VOCs) are used in a wider range of applications than ever before, including factories, residences, and other public facilities, and their monitoring has thus become increasingly necessary [1]. According to the World Health Organization, VOCs are a general term for volatile organic compounds with a melting point below room temperature and a boiling point between 50 °C and 260 °C, which are also typical atmospheric pollutants [2]. In gas sensors, metal oxide semiconductor (MOS) materials, including p-type and n-type materials, have attracted increasing research interest as VOC-sensor materials due to their satisfactory properties such as high sensitivity, small size, long-term stability, and low cost [2,3]. To improve the gas-sensing performance of MOS materials, common methods such as noble metal catalysis, doping, and heterojunction have been explored. Among these methods, doping is an effective and direct approach to enhance the sensing performance, given the complexity of heterojunction formation and the high cost of precious metals [3].

Compared to single MOSs, perovskite oxides with ABO_3_ structures form more oxygen vacancies, which are beneficial to gas-sensing, and the stable initial structure of perovskite oxides is maintained though the doping of transition metals on A or B sites [3,4]. Typically, transition metal cations with varying valence states and atomic radii can partially occupy the B-site. If only the change in valence state is considered, there are two different scenarios: first, if the valence state of the doped cation is lower than that of the cation on the initial B-site, the number of oxygen vacancies increases, which is due to the difference in their oxidation states; and second, if the valence state of the doped cation is higher than that of the cation on the B-site, lattice distortions lead to an increase in the number of oxygen vacancies [5].

Rare earth perovskite oxides with ABO_3_ structures exhibit excellent gas-sensing performance for VOCs after doping. For example, Changlin Xiao et al. lowered the detection limit of formaldehyde gas-sensing sensors to 1 ppb by utilizing the porous structure of In-doped LaFeO_3_ [6]; Jiaqian Gu et al. remarkably enhanced the performance of LaFeO_3_ for sensing n-butanol after Co doping, especially in terms of higher sensitivity, lower detection limit, etc. [7]. Meanwhile, triethylamine (TEA) has a boiling point of 89.5 °C under normal atmospheric pressure and its molecule contains hydrogen and carbon atoms, thus classifying it as a volatile organic compound (VOC) [8]. TEA, a widely used VOC, holds significant importance in various fields including organic solvents, multifunctional catalysts, anticorrosive agents, synthetic dyes, and surfactants. Additionally, it exhibits characteristics conducive to potential commercialization, low cost, and favorable physicochemical properties [1,9,10]. Generally, TEA is released from decaying fish and shellfish, which makes TEA a criterion for judging the freshness of seafood. Extended exposure to TEA, known for its pungent odor, can harm the human body by irritating the skin, mucous membranes, and nervous system. This irritation can result in respiratory discomfort, pulmonary edema, skin and mucous membrane damage, and in severe cases, death [11,12,13,14]. The National Institute for Occupational Safety and Health (NIOSH) recommends that indoor TEA concentration should not surpass 10 ppm for safety [8,15]. Additionally, exposure of TEA mixtures to open flames and high temperatures can lead to fires or explosions [1,10]. Therefore, exploring TEA gas-sensitive materials and devices is crucial for ensuring human health and safety.

According to previous studies by other researchers, there are indications that Mo doping in catalysts can elevate the oxygen vacancies. For example, Jie Zhang et al. prepared monoatomic Mo-doped TiO_2_ catalysts with abundant oxygen vacancies using a hydrothermal method to explore the effect of Mo doping and the increase of oxygen vacancies on the selective catalytic reduction (SCR) of NO_x_ [16]; Ziteng Ren et al. also prepared Zn_2_SnO_4_ with transition metal Mo doping by a hydrothermal method and demonstrated experimentally that Mo doping induced an increase in oxygen vacancies, enhanced the ability to capture light, improved the separation and migration of photo-generated carriers, and ultimately facilitated the activation of formaldehyde, which resulted in a significant increase in the formaldehyde photo-oxidation efficiency of the doped Zn_2_SnO_4_ [17]; Jiayu Li et al. doped Mo onto the surface of W_18_O_49_ by substituting W sites, which adjusted the atomic and electronic structures on the surface of the W_18_O_49_ catalyst, formed more oxygen vacancies, and ultimately significantly improved the catalytic activity [18]. Consequently, in this work, LaFeO_3_ gas-sensing materials with varying Mo-doping ratios were fabricated using the sol–gel method. The Mo doping significantly enhanced the gas-sensing response towards TEA gas. Via mechanism analyses, this work provides a strategy and reference for preparing high-performance perovskite metal oxide gas-sensitive materials.

## 2. Experiment

### 2.1. Preparation of Mo-Doped LaFeO_3_ Samples

LaFeO_3_ samples with different Mo-doping ratios were prepared via the sol–gel method. The doping ratios were 0%, 1%, 2%, 4%, and 8%. The sample preparation involved the following steps: 1 mmol (433 mg) of lanthanum nitrate hexahydrate (La(NO_3_)_3_·6H_2_O) was used for each group. Ferric nitrate nonahydrate (Fe(NO_3_)_3_·9H_2_O) was added in amounts of 1 mmol, 0.99 mmol, 0.98 mmol, 0.96 mmol, and 0.92 mmol (corresponding to 404 mg, 400 mg, 396 mg, 387.8 mg, and 371.68 mg, respectively) and ammonium molybdate tetrahydrate ((NH_4_)_6_Mo_7_O_24_·4H_2_O) was added at 0, 0.01, 0.02, 0.04, 0.08 mmol (0, 1.8, 3.53, 7.06, 14.1 mg) in that order. For each set of weighed samples, 3 mL of deionized water and 2 mmol of citric acid (420 mg) were sequentially added. The resulting mixtures were stirred on a magnetic stirrer for 15 min and then dried in an oven at 60 °C to form brown gels. Subsequently, the gels were left to dry overnight. The dried samples then underwent calcination in a muffle furnace at 750 °C for 5 h, with a heating rate of 5 °C/min.

### 2.2. Characterizations of Gas-Sensitive Materials

X-ray diffraction (XRD) was employed to analyze the structural information, crystal type, and grain size of the samples. The diffractometer model used in this study was a SHIMADZU XRD-7000S (SHIMADZU, Kyoto, Japan), with a scanning 2*θ* angle range of 5–80° and a scanning speed of 5°/min. For microscopic morphological observation of the synthetic materials, a field-emission scanning electron microscope (SEM) was utilized. Specifically, the model of SEM employed in this study was the FEI Nova NanoSEM450 (FEI, Hillsboro, OR, USA). High-purity ethanol, clean silicon wafers, and conductive adhesive were prepared for sample mounting, and the accelerating voltage was set to 18 kV. Nitrogen adsorption–desorption isotherm analysis was conducted to determine the specific surface area, pore volume, and pore size distribution of the samples. A fully automated physical adsorption instrument, the Quantachrome Autosorb-iQ-C (Quantachrome Instruments, Boynton, FL, USA) was utilized for this purpose. Prior to testing, cleaned BET tubes and a specific quantity of liquid nitrogen were prepared. Furthermore, X-ray photoelectron spectroscopy (XPS) was employed to ascertain the chemical composition, elemental valence, and percentage of the target sample surface. The X-ray photoelectron spectrometer used in this study was an ESCALABTM250Xi, manufactured by ThermoFisher, Waltham, MA, USA.

### 2.3. Preparation of Gas-Sensitive Elements

The sensing material and deionized water were thoroughly ground in a mortar to achieve a uniform paste. Subsequently, the paste was evenly applied to the alumina ceramic tube using a small brush, ensuring complete coverage of the tube at both ends of the gold electrodes. Following this, the coated gas-sensing device underwent a 6 h drying process in an oven set at 200 °C. Lastly, the dried gas-sensing device was affixed to the four electrodes of a hexagonal base, with a Ni–Cr alloy heating wire inserted into the alumina ceramic tube and welded onto the two electrodes of the hexagonal base.

### 2.4. Gas-Sensing Test

The sensing performance of the gas sensors was tested using a CGS-8 Intelligent Gas Sensitivity Analysis System (Beijing Elite Technology Co., Ltd., Beijing, China). Prior to the formal test, the prepared gas sensors were placed on the test instrument to undergo aging at 200 °C for 10 h.

The gas sensors were subsequently tested using the static gas distribution method. The details of the gas-sensing performance test were as follows. Aged gas sensors were inserted into the device sockets and covered with a closed organic glass mask. Then, the test instrument was turned on in order to record the surrounding temperature and humidity and the operating currents were set to control the heating temperature. After covering the organic glass mask, a specific amount of liquid target gas was drawn with a syringe. Subsequently, the gaseous target gas was injected into the gas chamber through the injection holes. The change in sensor resistance in the presence of the target gas could then be observed. According to the semiconductor type (p-type) of the synthetic gas-sensing materials described in this paper, the response value of the sensors towards reducing gases during the test was defined by the following formula (Equation (1)):

(1) $$Response=\frac{R_{g}}{R_{a}}$$

where *R_a_* and *R_g_* are the resistances of the sensing device in air and in the target gas, respectively. The response and recovery time was defined as the time required for the change in resistance to reach 90% of the total change in resistance after exposure to or departure from the target gas.

The conversion of the test gas concentration to the corresponding liquid volume is given below (Equation (2)):

(2) $$C=\frac{22.4\times Ø\times \rho \times V_{1}}{M\times V_{2}}\times 1000$$

where *C* (ppm) is the concentration of the target gas, *Φ* the purity of the liquid, *ρ* (g/mL) the density of the liquid, *V*_1_ (μL) the volume of the liquid, *V*_2_ (L) the volume of the gas chamber, and *M* (g/mol) the relative molecular mass of the target gas.

The ambient humidity was adjusted using a humidifier and recorded using the hygrometer included in the test instrument.

## 3. Results and Discussion

### 3.1. Characterizations of Sensing Materials

**Figure 1:**
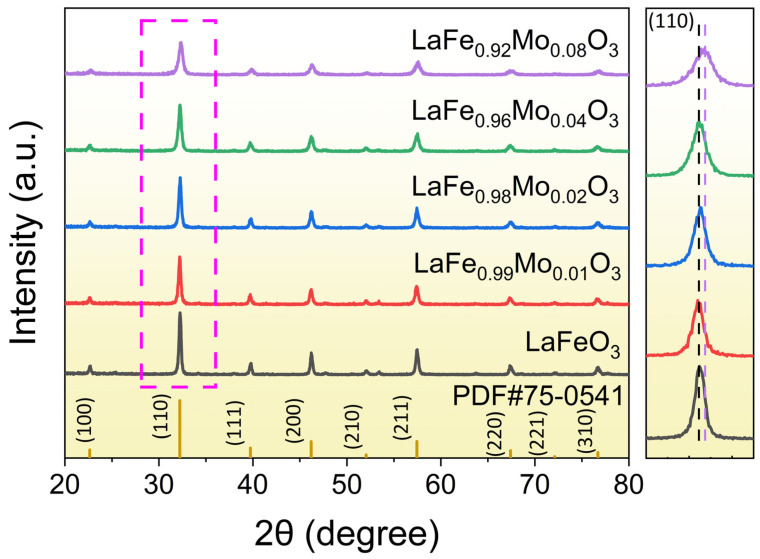
The XRD patterns of LaFe_1−δ_Mo_δ_O_3_ materials.

As shown in Figure 1, the crystal structures of the prepared samples were analyzed using XRD. In all samples, characteristic peaks at (100), (110), (111), (200), (210), (211), (220), (221), and (310) were observed, consistent with the orthorhombic LaFeO_3_ crystal phase (PDF#75-0541). In the XRD patterns, no additional diffraction peaks associated with impurities of Mo were observed. Simultaneously, Mo^6+^ replaced part of the Fe^3+^ and the radius of Mo^6+^ (0.59 Å) was smaller than that of Fe^3+^ (0.645 Å). By calculating the lattice parameters and crystallites sizes (Table 1), the increase in the Mo-doping ratio caused the lattice to shrink and the crystallites’ size to decrease. Therefore, as the percentage of Mo doping increased, the intensity of the (110) diffraction peak decreased while shifting towards higher 2*θ* values. These observations strongly suggested the successful incorporation of Mo into the crystal structure of LaFeO_3_, rather than the formation of a distinct phase [19].

**Figure 2:**
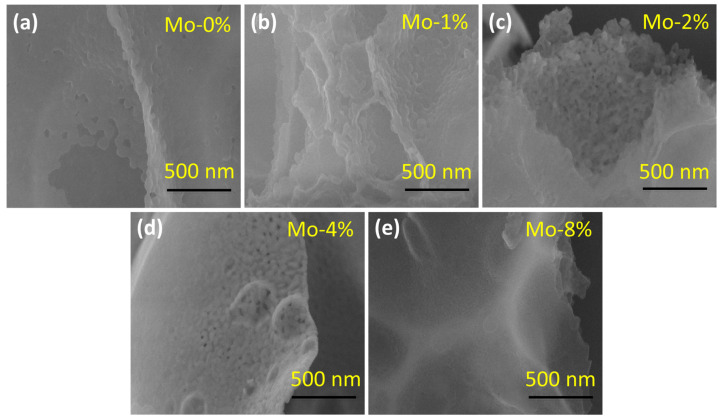
SEM images of LaFe_1−δ_Mo_δ_O_3_: (**a**) LaFeO_3_; (**b**) LaFe_0.99_Mo_0.01_O_3_; (**c**) LaFe_0.98_Mo_0.02_O_3_; (**d**) LaFe_0.96_Mo_0.04_O_3_; (**e**) LaFe_0.92_Mo_0.08_O_3_.

The morphology of the prepared samples was examined using SEM, as depicted in Figure 2. All the samples showed a sheet structure. With the increase in the Mo-doping ratio, the smooth surface of the samples gradually became rough and porous, and this phenomenon was especially obvious in the samples with 2% and 4% doping ratio of Mo (LaFe_0.98_Mo_0.02_O_3_, LaFe_0.96_Mo_0.04_O_3_, respectively). The porous structure was able to expose more active sites, promoting the diffusion of the target gas and accelerating the adsorption–desorption rate of the gas [20].

**Figure 3:**
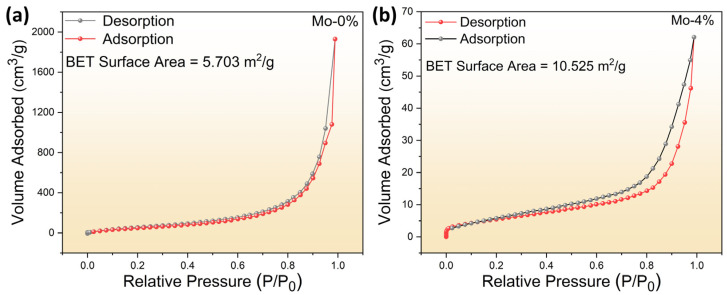
N_2_ adsorption–desorption isotherms of (**a**) LaFeO_3_ and (**b**) LaFe_0.96_Mo_0.04_O_3_.

In order to calculate the specific surface area of the prepared materials, we examined the N_2_ adsorption–desorption isotherms of LaFe_1−δ_Mo_δ_O_3_ samples as shown in Figure 3. The calculated specific surface areas of pure LaFeO_3_ and LaFe_0.96_Mo_0.04_O_3_ were 5.703 and 10.525 m^2^/g, respectively. The results showed that the specific surface area of LaFe_0.96_Mo_0.04_O_3_ was almost twice that of pure LaFeO_3_. A larger specific surface area is expected to improve the gas-sensing performance by exposing more active sites to oxygen target gas molecules.

**Figure 4:**
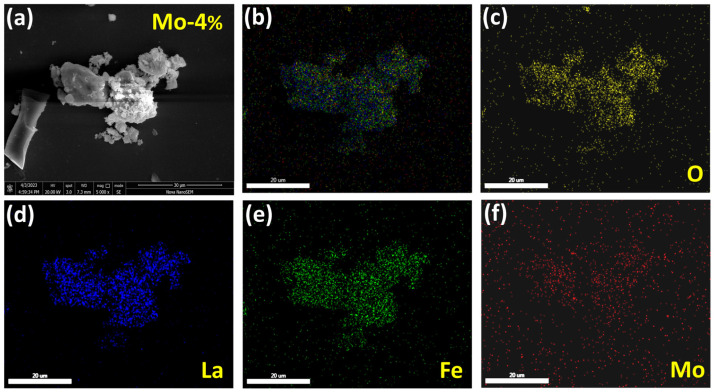
(**a**) SEM image, and (**b**–**f**) EDS images of LaFe_0.96_Mo_0.04_O_3_.

The elemental mapping images (Figure 4) clearly demonstrate the harmonious coexistence and the homogeneous distribution of La, Fe, O, and Mo atoms within the 4% Mo-doped LaFeO_3_ samples, providing additional confirmation of the successful and uniform incorporation of the Mo element in the material.

### 3.2. Gas-Sensing Performance

The gas-sensing properties of undoped and Mo-doped LaFeO_3_ were examined to evaluate the effect of Mo doping on LaFeO_3_.

**Figure 5:**
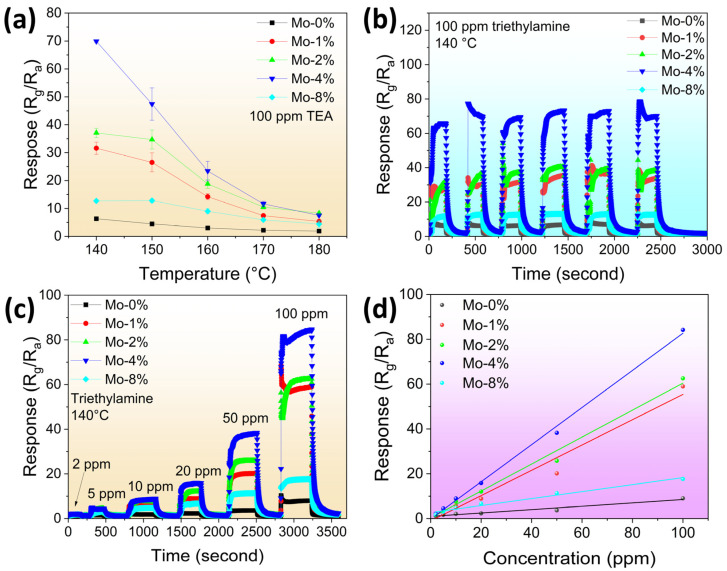
(**a**) LaFe_1−δ_Mo_δ_O_3_ sensors’ response curves versus temperature to 100 ppm TEA gas; (**b**) cyclic stability of LaFe_1−δ_Mo_δ_O_3_ sensors to 100 ppm TEA gas at 140 °C; (**c**) dynamic response–recovery curves of LaFe_1−δ_Mo_δ_O_3_ sensors to 2–100 ppm TEA gas at 140 °C; (**d**) linear fitting curves of LaFe_1−δ_Mo_δ_O_3_ sensors at 140 °C for the TEA response values in the concentration range of 2–100 ppm.

The operating temperature is a crucial parameter that influences the performance of gas sensing, since adequate thermal energy is generally necessary for the chemical adsorption and reaction of the test gas on the surface of the sensing material. In order to ascertain the optimal Mo-doping ratio and explore the correlation between the sensing response and the ideal operating temperature, samples of LaFeO_3_ doped with 0%, 1%, 2%, 4%, and 8% Mo were subjected to operation within the temperature range of 140–180 °C, as illustrated in Figure 5a. Evidently, the response of all the samples decreased with increasing temperature, so 140 °C was selected as the working temperature. Notably, the response of the 4% Mo-doped LaFeO_3_ sensor to 100 ppm TEA reached 70, a value approximately 11 times greater than that of pure LaFeO_3_ and greater than the other doping conditions. The cyclic reproducibility of the LaFeO_3_ sensors with different Mo-doping ratios was demonstrated across six reversible cycles at an operating temperature of 140 °C and 100 ppm TEA atmosphere, as displayed in Figure 5b. It is noteworthy that all sensors showed good reproducibility with relative standard deviations of 3.55%, 10.09%, 7.56%, 4.37%, and 5.31% for 0%, 1%, 2%, 4%, and 8% Mo-doped LaFeO_3_ samples, respectively.

In Figure 5c, the dynamic transient curves depict the responses of five sensors to varying concentrations of TEA gas, ranging from 2 ppm to 100 ppm, at a temperature of 140 °C. The observed trend indicated that the sensor’s response value steadily increased with the escalation of TEA concentration. The sensing characteristics of these sensors were found to be closely associated with the level of Mo-doping ratio in the sensing material. In order to determine the linearity of the response values with respect to the dependence on TEA concentration, relationships between the response values of the LaFe_1−δ_Mo_δ_O_3_ sensor are shown in Figure 5d. Within the 2–100 ppm range of the test gas (TEA), the 4% Mo-doped LaFeO_3_ sensor showed the best sensitivity with the maximum slope (0.8284) among the different doping ratios, and a remarkably robust linear correlation between the response and the concentration was apparent (R^2^ = 0.99736).

Obviously, the 4% Mo-doping ratio in the LaFeO_3_ sensor provided an optimal sensing performance. To further evaluate the utilization potentiality of 4% Mo-doped LaFeO_3_, tests to determine the response/recovery times, selectivity, stability, and the influence of humidity were performed.

**Figure 6:**
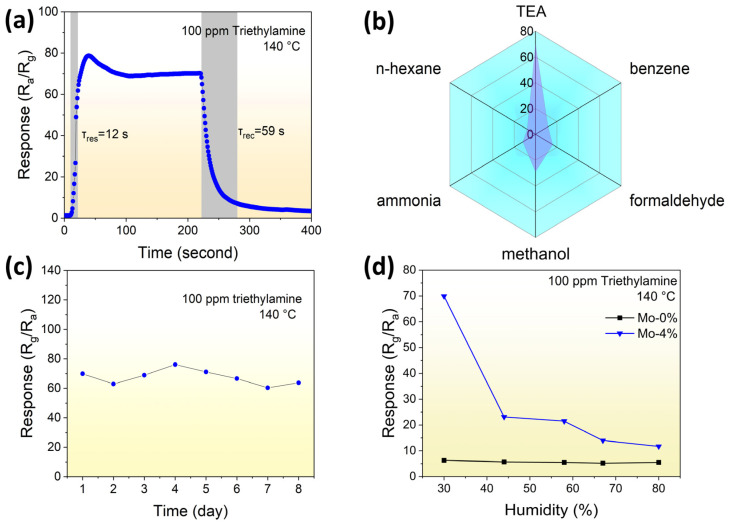
(**a**) Response/recovery time of LaFe_0.96_Mo_0.04_O_3_ to 100 ppm TEA; (**b**) response of the LaFe_0.96_Mo_0.04_O_3_ sensor to 100 ppm TEA, n-hexane, formaldehyde, ammonia, and methanol gases at 140 °C; (**c**) long-term stability and (**d**) relative humidity stability of the LaFeO_3_ and LaFe_0.96_Mo_0.04_O_3_ sensors.

The response time and recovery time of the 4% Mo-doped LaFeO_3_ sample are shown in Figure 6a. It can be seen that the 4% Mo-doped LaFeO_3_ sensor exhibited a relatively rapid response sensitivity of about 12 s. It is worth noting that the recovery time of the sensor is considerably longer compared with the response time. This disparity in time can be attributed to the sluggish nature of the surface reactions, such as oxygen adsorption, dissociation, and ionization processes.

To evaluate selectivity, we tested the response of the 4% Mo-doped LaFeO_3_ sensor in an atmosphere of TEA, n-hexane, formaldehyde, ammonia, and methanol (Figure 6b). The gases were tested at a concentration of 100 ppm and an operating temperature of 140 °C. The results indicated that the response of the 4% Mo-doped LaFeO_3_ sensor to 100 ppm TEA at 140 °C was 69.9, which was more than twice as high as the response to the other tested gases (7.84 for n-hexane, 16.0 for formaldehyde, 11.9 for ammonia, 29.0 for methanol, and 11.8 for benzene). This selectivity may have been related to the different activation energies of adsorption, desorption, and reaction of the target gas on the surface of the sensing material [21,22]. The selectivity of a sensor for certain gas molecules can be caused by the different reactivity of the sensor for different bond energies. The bond energies of H-H, N-O, N-H, O-H, C-N, and C=O are 436, 466, 391, 458.8, 307, and 789.9 kJ/mol, respectively. The weak bonding strength of C-N in TEA plays a role in elucidating the heightened reactivity of TEA molecules on the surface of 4% Mo-doped LaFeO_3_ sensors [23,24].

As shown in Figure 6c, the long-term stability of the 4% Mo-doped LaFeO_3_ sensor was demonstrated by repeating the test at 140 °C for 8 days with 100 ppm TEA. It was observed that the response of the 4% Mo-doped LaFeO_3_ sensor exhibited a variation of only 7.58% and did not appear to weaken or oscillate significantly, indicating good long-term stability.

To explore the dependence of the sensing materials on relative humidity, the range of relative humidity was 30–80%; the test results are shown in Figure 6d. It can be seen that the response of the 4% Mo-LaFeO_3_ sensor decreased significantly in the range of 30–45%RH but in the range of 45–80%RH, the response of the 4% Mo-LaFeO_3_ sensor was maintained above 20 and remained stable. The reasons for the significantly lower response of LaFeO_3_ sensors in the range of 30–45%RH may have been as follows.

When water vapor was introduced into the test chamber, adsorbed oxygen and water molecules reacted to form hydroxyl groups (Equation (3)), which hindered the reaction of the target gas with chemisorbed oxygen [25]. The absorption of water molecules on the surface of the sensor material prevented the absorption of oxygen and reduced the concentration of oxygen species adsorbed on the surface of the material [26], which further reduced the gas-sensing response.

(3) $$H_{2}O+O_{\left(ads\right)}^{-}\left(O_{2\left(ads\right)}^{-}\right)\to 2\left(OH^{-}\right)+\left(2\right)e^{-}$$

The reasons for the stabilization of the response of the 4% Mo-LaFeO_3_ sensor with increasing humidity in the range of 45–80%RH may have been as follows.

Water molecules consumed oxygen vacancies to form hydroxyl groups, and the hydroxyl groups formed also seized active sites of oxygen for adsorbtion on the material surface. When the relative humidity of the environment reached a certain level, almost all of the original active sites for the adsorption of the oxygen on the surface of the sensor material were seized by the hydroxyl groups. Meanwhile, the concentration of the oxygen on the surface tended to be stable, which in turn stabilized the response of the sensor material to TEA.

### 3.3. Gas-Sensing Mechanism

Catalytic activity of the chalcogenide oxide ABO_3_ is primarily governed by the B-site. In the case of LaFeO_3_, gas molecules, such as TEA, tend to preferentially adsorb on the Fe-site during the sensor response [22,27,28]. The ionic radii of Fe and Mo are Fe^3+^ (0.645 Å) and Mo^6+^ (0.59 Å) [29]. In this study, the radius of the Mo^6+^ ion (0.59 Å) was comparable to that of the Fe^3+^ ion (0.645 Å) and significantly smaller than that of the La^3+^ ion (1.36 Å). Due to this size compatibility, Mo^6+^ ions tended to preferentially occupy the octahedral sites rather than the cuboctahedral voids in the crystal lattice [30,31]. As a result, in the context of LaFeO_3_, Mo^6+^ ions were doped into the Fe site (B site).

**Figure 7:**
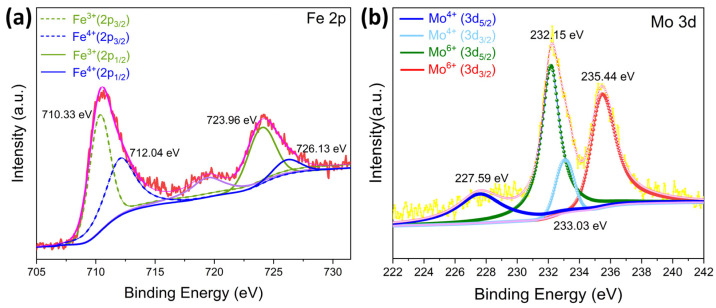
XPS patterns of LaFe_0.96_Mo_0.04_O_3_: (**a**) Fe 2p; (**b**) Mo 3d.

Given the superior gas-sensing performance of 4% Mo-doped LaFeO_3_ and the significant impact of the material surface state on gas-sensing characteristics, it is crucial to prioritize the analysis of the surface state of this specific composition. The surface elemental composition and states of the 4% Mo-doped LaFeO_3_ sample were evaluated through XPS analysis. Binding energy shifts were calibrated with respect to C 1s (284.8 eV), serving as a reference standard. In Figure 7a, the peaks of Fe 2p_3/2_ and Fe 2p_1/2_ are centered at 710.35 and 723.78 eV, respectively, indicating the Fe^3+^ states in orthorhombic LaFeO_3_, suggesting that Fe predominantly existed as Fe^3+^. Furthermore, Figure 7a shows that smaller portions of the characteristic peaks of Fe 2p_3/2_ and Fe 2p_1/2_ were located at 712.60 and 725.4 eV, corresponding to the Fe^4+^ state, which implies that a small portion of Fe existed in the form of Fe^4+^. For ideal LaFeO_3_, all Fe elements should exist as Fe^3+^. However, from the XPS analysis, it was observed that the Fe element in the sample existed as both Fe^3+^ and Fe^4+^. This was due firstly to the insufficiency of the positive charge induced, with the conversion of Fe^3+^ to Fe^4+^ making up for the insufficiency of positive charge and maintaining the electroneutrality of the material; secondly, the oxygen adsorption led to the adsorbed oxygen capturing electrons from the Fe^3+^, which resulted in the formation of Fe^4+^ [32]. In Figure 7b, the XPS spectrum of the Mo 3d orbitals displays two similar characteristic peaks at 232.15 and 235.44 eV, corresponding to the Mo 3d_5/2_ and Mo 3d_3/2_ orbitals, respectively. This suggests that Mo primarily existed in the form of Mo^6+^. Additionally, the sample had characteristic peaks with lower intensities located at 227.59 eV and 233.03 eV, corresponding to the Mo^4+^ state, which implies that a small portion of Mo existed in the form of Mo^4+^. The appearance of Mo^4+^ may have been due to the abundant oxygen vacancies in the material [33]. It is worth noting that Mo^6+^ exhibits stronger reducing properties compared with Fe^3+^ [34], rendering the gas-sensing material more prone to react with atmospheric oxygen. This reactivity, in turn, promotes the chemisorption of oxygen.

**Figure 8:**
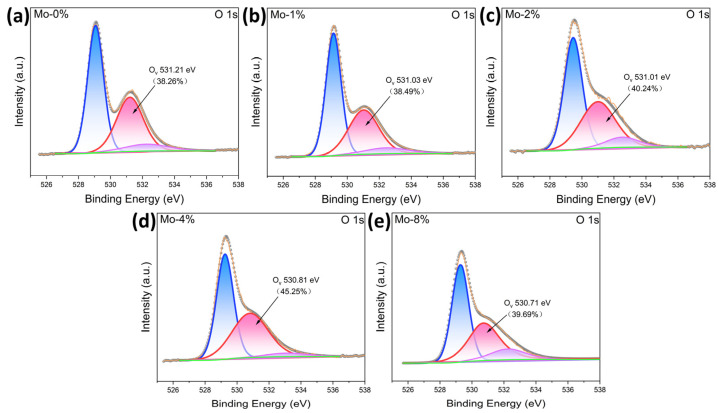
O 1s orbital XPS patterns of LaFe_1−δ_Mo_δ_O_3_ with doping ratios of (**a**) LaFeO_3_; (**b**) LaFe_0.99_Mo_0.01_O_3_; (**c**) LaFe_0.98_Mo_0.02_O_3_; (**d**) LaFe_0.96_Mo_0.04_O_3_; (**e**) LaFe_0.92_Mo_0.08_O_3_.

As depicted in Figure 8, using the Gaussian function fitting method, three main peaks at ca. 529, 530, and 533 eV were observed in the O 1s spectra, matching to lattice oxygen (O_L_), oxygen vacancies (O_V_s), and adsorbed oxygen (O_ads_), respectively [35,36,37]. Figure 8 illustrates the percentage of oxygen vacancies in the samples with varying Mo-doping ratios. Notably, the oxygen vacancy content increased with the doping amount compared with the pure LaFeO_3_ sample. However, the oxygen vacancy ratio experienced a rapid decrease when the doping amount exceeded 4%. Among the samples, the 4% Mo-doped LaFeO_3_ sample exhibited the highest oxygen vacancy ratio, reaching 45.24%. This value was approximately 20% higher than that of the pure samples. Abundant oxygen vacancies can significantly reduce the adsorption energy [38] and significantly increase the adsorption of gas molecules on the surface. The increase in oxygen vacancies promotes the adsorption of oxygen molecules and enhances oxygen mobility, thereby expediting the redox process. The enhancement of oxygen vacancies plays a pivotal role in improving the gas-sensing performance [5].

Based on Figure 8d,e, the oxygen vacancy content of the 8% Mo-doped LaFeO_3_ sample decreased to 39.69% in comparison to that of the 4% Mo-doped LaFeO_3_ sample. This reduction in the oxygen vacancy ratio was identified as the primary cause for the lower sensitivity of the 8% Mo-doped LaFeO_3_ sample. Conversely, the 4% Mo-doped LaFeO_3_ sample exhibited the highest oxygen vacancy ratio among all samples, which accelerated the rate of redox reactions of the target gas molecules. Simultaneously, the porous structure of the 4% Mo-doped LaFeO_3_ sample also played a complementary role by increasing the number of active sites for the adsorption of oxygen, facilitating reactions with the target gas molecules. Consequently, the gas-sensitivity performance of the 4% Mo-doped LaFeO_3_ sample surpassed that of the LaFeO_3_ materials with other Mo-doping ratios.

**Figure 9:**
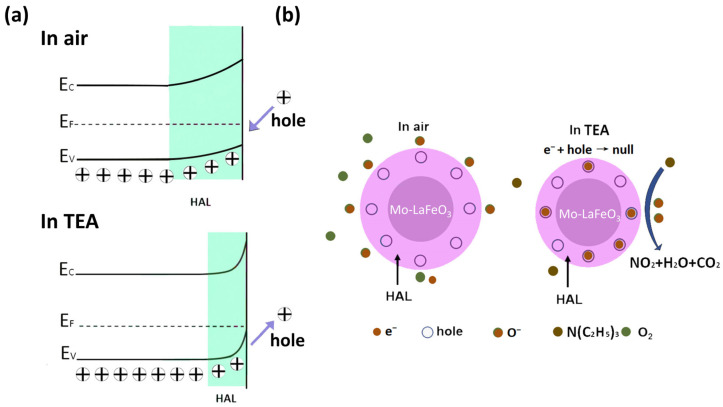
(**a**) Energy band structure diagram and (**b**) mechanism diagram of Mo-LaFeO_3_ sensing material for TEA gas.

The usual process of the gas-sensing reaction occurs as follows: when Mo-doped LaFeO_3_ is exposed to air, oxygen molecules are adsorbed on the surface active sites of the gas sensor and capture electrons from the conduction band of LaFeO_3_, leading to the formation of differently chemisorbed oxygen on the surface of the sensing material under different temperature conditions. This process is accompanied by an upward bending of the energy bands (Figure 9a). The adsorption of oxygen can be described as follows (Equations (4)–(6)) [39,40]:

(4) $$O_{2\left(gas\right)}\;\to \;O_{2\left(ads\right)}$$

(5) $$O_{2\left(ads\right)}+e^{-}\to \;O_{2\left(ads\right)}^{-}$$

(6) $$O_{2\left(ads\right)}^{-}+e^{-}\to \;{2O}_{\left(ads\right)}^{-}$$

By trapping electrons from the conduction band of LaFeO_3_, the concentration of holes increases, leading to the formation of a hole accumulation layer (HAL) near the surface of the LaFeO_3_. This reduces the resistance of the sensing material [41,42]. When the LaFeO_3_ sensor is exposed to TEA gas, the gas molecules of interest undergo a chemical reaction with the oxygen that is chemisorbed on the sensor’s surface. This reaction leads to the liberation of trapped electrons, which are then released back into the conduction band of the sensing material. The recombination of these electrons with the holes results in a reduction in the HAL thickness and an elevation in the resistance of the sensing material, as depicted in Figure 9b. This process can be represented as follows (Equations (7) and (8)) [39]:

(7) $${{2N\left(C_{2}H_{5}\right)}_{3}}_{\left(ads\right)}+{{43O}^{-}}_{\left(ads\right)}\;\to \;{2NO}_{2}+{15H}_{2}O+12CO_{2}+43e^{-}$$

(8) $$e^{-}+h\cdot \;\to \;null$$

Meantime, Equation (10) can be subdivided into the following equations (Equations (9)–(11)) [24]:

(9) $$\left(C_{2}H_{5}\right)N\to 3C_{2}H_{4}+NH_{3}$$

(10) $$C_{2}H_{4}+6O^{-}\to 2CO_{2}+2H_{2}O+6e^{-}$$

(11) $$NH_{3}+7O^{-}\to 2NO_{2}+3H_{2}O+7e^{-}$$

The high sensitivity and selectivity of Mo-doped LaFeO_3_ sensing materials for TEA gases can be attributed to the following reasons:

(1) In the LaFeO_3_ lattice, the doping of Mo leads to distortion of the LaFeO_3_ lattice due to the difference in radii, which introduces defects. The presence of lattice defects provides more activation sites for the sensing reaction, which helps to improve the response value and selectivity [41];
(2) The synergistic effect of morphology and surface composition is threefold. Firstly, the porous nanosheet structure, with its large specific surface area, exposes more reactive active sites, promoting the diffusion of TEA molecules and accelerating the adsorption–desorption rate of the target gas. Secondly, a higher content of adsorbed oxygen on the surface leads to the exposure of more active sites. Finally, the presence of oxygen vacancies on the material’s surface alters the electronic state of the metal cations and provides active sites for the gas-sensing process [32].

## 4. Conclusions

In this work, LaFe_1−δ_Mo_δ_O_3_ (δ = 0, 0.01, 0.02, 0.04, and 0.08) semiconductor nanomaterials were synthesized by the sol–gel method. The XRD patterns indicated an orthorhombic perovskite crystal phase and the SEM images showed the nanostructure of the materials. The (110) diffraction peak shifted towards higher 2*θ* values with increased Mo doping, with lattice distortion due to the Mo doping. Mo doping improved the gas-sensing properties of the materials, most notably in the sample with 4% Mo doping, which exhibited a response value approximately 11 times greater than that of pure LaFeO_3_. The 4% Mo-doped LaFeO_3_ sensor also showed a low working temperature of 140 °C, good selectivity towards TEA gas, and a perfect linear correlation between the response and the concentration (R^2^ = 0.99736). The remarkable gas-sensing performance of 4% Mo-doped LaFeO_3_ can be attributed to the lattice distortion, the increased oxygen vacancies, and the porous morphology. This project can contribute to the development of an innovative, cost-effective gas sensor capable of accurately monitoring triethylamine gas concentrations to safeguard human health and safety.

## Figures and Tables

**Table 1:** The lattice parameters and crystallite sizes of LaFe_1−δ_Mo_δ_O_3_ materials.

Sample	a (Å)	b (Å)	c (Å)	Volume (Å^3^)	Crystallite Size (Å)
LaFeO_3_	5.55	7.84	5.55	241.4916	345
LaFe_0.99_Mo_0.01_O_3_	5.55	7.87	5.54	241.9789	291
LaFe_0.98_Mo_0.02_O_3_	5.55	7.85	5.54	241.3640	263
LaFe_0.96_Mo_0.04_O_3_	5.54	7.86	5.54	241.2360	228
LaFe_0.92_Mo_0.02_O_3_	3.91	3.91	3.95	60.3880	168

## Data Availability

The authors confirm that the data supporting the findings of this study are available within the article.
